# Engineered Gold-Based Nanomaterials: Morphologies and Functionalities in Biomedical Applications. A Mini Review

**DOI:** 10.3390/bioengineering6020053

**Published:** 2019-06-10

**Authors:** Iole Venditti

**Affiliations:** Department of Sciences, University of Roma Tre, via della Vasca Navale 79, 00146 Rome, Italy; iole.venditti@uniroma3.it; Tel.: +39-06-5733-3388

**Keywords:** gold nanoparticles, gold nanorods, gold nanostars, gold nanocubes, gold hollow nanoparticles, gold nanocapsules, gold nanocages, therapy, diagnostics, theranostics, drug delivery, gene delivery, sensing, imaging

## Abstract

In the last decade, several engineered gold-based nanomaterials, such as spheres, rods, stars, cubes, hollow particles, and nanocapsules have been widely explored in biomedical fields, in particular in therapy and diagnostics. As well as different shapes and dimensions, these materials may, on their surfaces, have specific functionalizations to improve their capability as sensors or in drug loading and controlled release, and/or particular cell receptors ligands, in order to get a definite targeting. In this review, the up-to-date progress will be illustrated regarding morphologies, sizes and functionalizations, mostly used to obtain an improved performance of nanomaterials in biomedicine. Many suggestions are presented to organize and compare the numerous and heterogeneous experimental data, such as the most important chemical-physical parameters, which guide and control the interaction between the gold surface and biological environment. The purpose of all this is to offer the readers an overview of the most noteworthy progress and challenges in this research field.

## 1. Introduction

**Figure 1:**
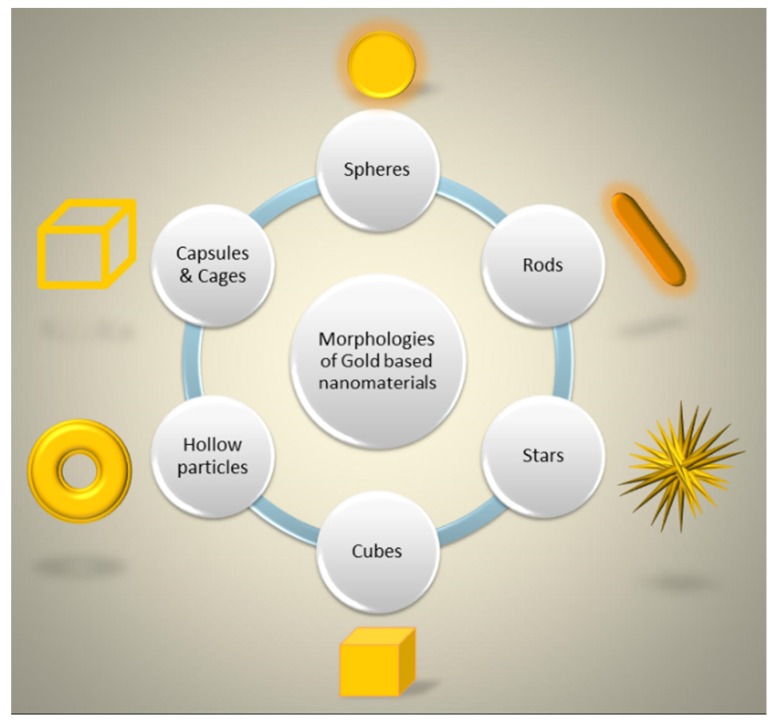
Schematic representation of the main morphologies of gold-based nanomaterials used in biomedical applications.

In the last decade, nanotechnology allowed for an improvement of materials properties in many application fields, such as sensing [1,2,3,4,5,6,7,8,9], optoelectronics [10,11,12,13,14,15], energy [16,17,18,19,20,21], catalysis [22,23,24] and biotechnology [25,26,27,28,29,30,31]. Gold was used to produce many different nanostructures through a bottom-up approach, such as spheres, rods, stars, cubes, hollow nanoparticles, and nanocapsules, widely used in biomedicine and reported in a schematic way in Figure 1. These nanostructures showed remarkable chemical-physical properties, and often their superficial functionalizations allow a response to environmental changes, such as variations in the temperature, pH, light, and redox potentials [6,8,26,30,32,33,34,35]. This represented an amazing opportunity for their use in active therapies, as drug carriers, as theranostic agents, and as sensing materials [6,26,30,33,36,37,38,39]. In particular, localized surface plasmon resonance (LSPR) plays an important role in several nanotechnological applications. Electrons on the surface of noble metal nanoparticles, interacting with electromagnetic radiation, generate LSPR and, for this reason, metal nanoparticles produce strong extinction and scattering spectra, useful for many and different applications.

Other application examples are the visualization methods that use gold nanoparticles and confocal laser microscopy, which show great attractiveness in the biomedical and biosensing field [40]. In fact, several methods, such as fluorescence detection (confocal fluorescence microscopy) or resonance elastic or two-photon light scattering (resonance scattering confocal microscopy or two-photon luminescence confocal microscopy) resulted in notable confocal images [41,42]. The main advantage of this method is the background signal decrease and, at the same time, the enhancement of the contrast. Another popular method in biological imaging is represented by dark-field microscopy, in which the objects with a size under the resolution limit of a light microscope, induce light scattering. A new application was developed by American researchers at the El Sayed laboratory, based on the spherical gold nanoparticles (AuNPs) preferentially bonded to cancerous cells, as compared with binding to healthy cells, using AuNPs conjugated with antibodies specific to tumor antigens [43]. In these cases, resonance scattering dark-field microscopy is used to map a tumor with a high accuracy. In successive studies, gold nanorods, nanostars, and nanocages were used with the same aim [44,45]. Among others, some modern methods for biological imaging have recently been developed; they can be called biophotonic methods, and they study the light-biological matter interaction. Optical coherence tomography, X-ray and magneto-resonance tomography, photoacoustic microscopy and tomography, as well as fluorescence correlation microscopy, can be included as part of the biophotonic methods [46]. 

Among others, Raman imaging of surface enhanced Raman scattering (SERS) nanoparticles is an optical technique that offers an incomparable sensitivity (on the order of 10^−15^–10^−12^ M) and multiplexing abilities to the field of molecular imaging. Raman spectroscopy is due to the inelastic scattering of light upon interaction with a molecule, used to produce a sample fingerprint. Raman scattering has an inherently weak effect. However, if the incident photon loses or gains energy as it interacts with the molecule, this produces Stokes or anti-Stokes Raman scattering, respectively. SERS consists of the interaction of a Raman reporter with a roughened metal surface, which gives an electromagnetic enhancement of signal of the order of 10^4^ to 10^8^ over spontaneous Raman. It can be exploited for sensing and for diagnoses. The SERS technique is used to realize innovative probes that associate metallic nanoparticles with specific organic Raman reporter molecules. These SERS-active probes are used to indirectly sense the target molecules by using laser Raman spectrometry or SERS microscopy. Therefore, they show optical labeling functions like organic ones. These kinds of probes have a typical ultrasensitivity, as well as the multiplexing and quantitative skills of the SERS technique, and they show amazing potentials for bioanalysis [6,26,30].

Gold-based materials, with various dimensions and shapes, are also used in these methods, both for therapeutic and diagnostic applications [47]. Furthermore, several studies have been conducted to verify their low toxicity. Generally the results show how the toxicity and cytotoxicity of gold nanomaterials depend on the size and surface chemistry: they are mostly nontoxic after acute exposures, as long as the particles are around 4–5 nm in diameter [48,49,50], while particles larger than 5 nm can have toxic effects due to toxic surface coatings [51,52]. Often, acute toxicity can be attributed to the use of very high concentrations or specific cell type sensitivities [53,54].

In a wide panorama of innovative materials, this review tries to give an up-to-date view of the progress of nanosized gold-based materials. In fact, the last years have seen the creation of numerous morphologies, schematically summarized in six shapes in Figure 1, such as spheres (AuNPs), rods (AuNRs), stars (AuNSs), cubes (AuNCs), hollow particles (AuHNPs), and capsules and cages (AuNCaps and AuNcages), each one with advantages and critical issues. Moreover, the importance of surface specific chemistry has a crucial role in view of biomedical applications, and the multidisciplinary research activities take this point greatly into account. 

## 2. Engineered Gold-Based Nanomaterials 

The opportune choice of nanomaterials, in terms of the morphology, size and functionalization, allows the improvement of their performance, such as, for example, the sensitivity, selectivity, biocompatibility, traceability, drug loading, bioavailability, and controlled-targeted release. The current challenge is the introduction of many and different functionalizations on well-reproducible nanomaterials, with a specific defined morphology and size, with the aim of obtaining an engineered nanomaterial, i.e., a single object able to do multi-actions. Many gold-based nanomaterials have been developed for biomedical applications, due their unique chemical-physical properties [1]. In fact, they have an easy modifiable morphology/size/surface, modulable optical absorption and scattering, photothermal and photoacoustic efficiencies and wide surface/volume ratio, suitable for interaction with the environment [55,56,57,58]. For these materials, the surface chemistry is the main component of their biological targeting characteristics: when the nanoparticles come in contact with the cellular membranes surface, this can produce the internalization and intracellular localization [59,60]. Moreover, the gold surface can be functionalized and linked with biomolecules, such as enzymes, antibodies, DNA, and peptides to achieve a specific site [61,62].

Noble metal-based nanoparticles are also studied to develop new antibacterial systems. The increasing resistance of microbes to medicines induces researchers to develop new effective systems to fight them. The gold-based nanomaterials are used in two different antibacterial approaches: antibacterial photothermal therapy (APTT) and antibacterial photodynamic therapy (APDT). Both of these approaches are very interesting because they do not cause drug resistance. In APTT, the gold-based nanomaterials are photothermal agents (PTAs), i.e., they can transform light into thermal energy under appropriate radiation. For example, AuNRs and AuNSs are used in the APTT approach for biofilm disinfection via laser irradiation, generating localized hyperthermia to lyse bacteria [63,64,65]. The APDT approach is based on the production of reactive oxygen species (ROS), generated by irradiated photosensitizers, to kill bacteria. However, the antibacterial performance of APDT is lower for Gram-negative bacteria compared with that for Gram-positive bacteria, and the combination of APDT with other antibacterial methods is the best way to improve its efficacy. Therefore, hybrid silver-gold nanomaterials can be used in a synergistic way, due to the capability of silver nanoparticles (AgNPs) themselves to improve the generation of ROS under the visible region. Hybrid Au-Ag core-shell NRs are also developed to synergize the antibacterial effect due both to the plasmonic heating and to the release of Ag+ ions [65,66]. Of course, AuNPs can also act as vehicles for antibiotics, thus increasing their bactericidal effect. Several conjugate systems were designed, prepared and tested, properly functionalizing the surface of the AuNPs to bind one or more antibiotics. In these cases, AuNPs@antibiotics conjugates showed a higher antibacterial efficiency, compared to pure antibiotics and AuNPs on its own [67,68]. These hybrid and composite gold-based nanomaterials are under deep investigations for their use as potential therapy agents against bacterial infections [69].

As is well known, morphology plays a key role in gold-nanotechnology, especially in relation to the possibility of having materials with different symmetries (spheres, rods, and cubes) [1,2,3,4,12,24,28,70,71,72,73,74] or without a defined and reproducible symmetry (stars, hollow particles, and cages) [1,75,76,77,78,79,80,81,82,83,84]. Highly symmetrical particles are the most studied because of their low dispersity and synthetic reproducibility, which assure the repeatability of experiments and results. On the other hand, the non spherical and/or heterogeneous gold particles open new opportunities to enhance sensitivity. In fact, by generating local electromagnetic fields near particles, with sharp areas on their surface or in the cavities between two nanoparticles, the amplification of the biomolecular binding signal occurs. This phenomenon enhances the sensitivity of plasmon resonance to the local dielectric environment and produces a high scattering intensity in comparison with spherical shape particles with the same volume. Furthermore, the non-spherical morphologies present the possibility of having the plasmonic band in the near infrared (NIR) range. This is a great advantage because the range of ∼800 nm (NIR) is optimal for the best tissue penetration of light, and both AuNSs and AuNRs can be used. It is also necessary to stress that, although small AuNPs generally absorb at 500–600 nm, they can be easily conjugated to target biomolecules. In this way, they are easy internalized by tumor cells and, inside the tumor cell, they aggregate due to enzyme actions or/and by pH effects. This fact has two positive consequences: (i) small AuNPs shift their absorption into the NIR region; however, they are NIR transparent until they accumulate in tumor cells; (ii) the use of targeted small AuNPs offers a powerful method for discriminating NIR tumor therapy, reducing background heating in blood and non-targeted cells, and increasing specificity [2,8,10,48,85].

All of this also makes possible many significant applications for diagnostics, for example by means of dark-field microscopy. In general, different shapes and sizes offer advantages and disadvantages and may be more appropriate in some applications than others. Moreover, many and different functionalizations are introduced on the gold nanomaterials surface, such as amine, thiols, sulfonates, alcohols, esters, and acids. In Table 1, the morphologies, dimensions, and frequently surface functionalities of gold-based nanomaterials were reported for the common biomedical investigations. Naturally, several chemical preparation strategies are used, and the main ones will be illustrated in the following paragraphs.

**Figure 2:**
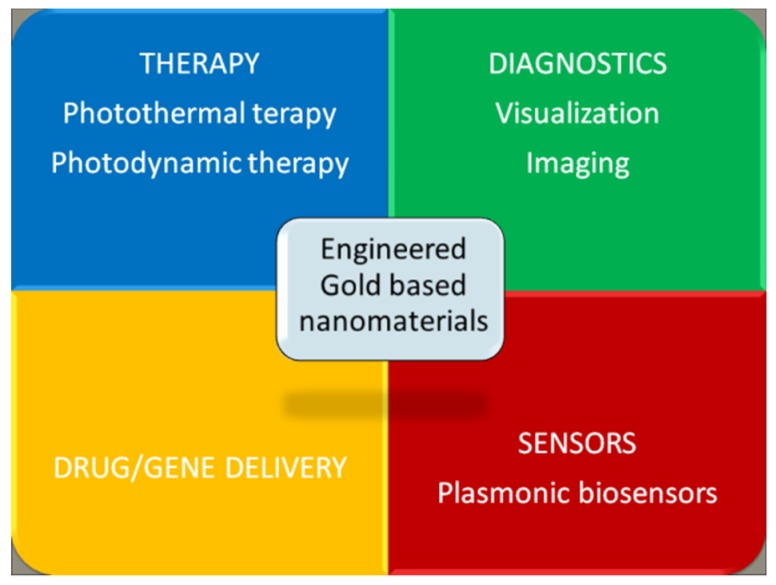
A schematic representation of the four main areas of biomedical applications for engineered gold-based nanomaterials.

This review shows the advantages and disadvantages of the different morphologies, considering the dimension, functionalization and bioapplication. In this regard, in the wide biomedicine field, four main areas of applications are typically identified: therapy, diagnostics, drug/gene delivery, and sensors, as schematically shown in Figure 2 [4,8,10,12,16,17,18,21,28,29,30,31,32,33,34,37,38,39,48,51,53,70,71,72,75,76,77,78,79,80,81,82,83,84,85,90,93,94,95,96,97,98,99,100,101,102,103,104,105,106,107,108,109,110,111,112,113,114].

In the therapy field, a new method in medical treatment is photothermal therapy (PTT). It can use AuNPs with a diameter of around 30–100 nm, radiated by laser pulses, in order to produce local warming-up, to induce the selective damaging of target cells. The laser irradiations with pulses allows for the regulation of cell inactivation using no-shocking methods. Moreover, nanoparticles make photothermal therapy realizable using optical tomography [115]. The PTT efficiency of AuNPs depends on their shape, dimensions, structure, and aggregation [116]. In fact, although AuNPs are ineffective in NIR, their aggregates can be very efficient (with interatomic distances below 10% of diameter) [117,118]. The small aggregates consisting of 30 nm particles enable the destruction of cancer cells at an intensity that is lower by a factor of 20 than that in the particle-free control. The use of AuNPs for PTT on chemotherapy-resistant type cancers is really promising, and nowadays many studies on the application of AuNPs, AuNRs and AuNcages in PTT are available [118,119,120,121,122,123]. Photodynamic therapy (PDT) is another new method applied both for oncological diseases and infectious diseases. In general, light-sensitive agents (photosensitizers) can be selectively accumulated in target tissues after their intravenous administration. Then, when opportune laser light radiates tissues, a heat release is produced, due to absorption. Furthermore, the photochemical generation of singlet oxygen and the formation of active radicals occur, and these species induce the necrosis and apoptosis of tumor cells. The main problem of PDT results from the long persistence of the photosensitizer in the body, which makes patients sensitive to light. Moreover, the use of dyes for the selective heating of tissues is characterized by a low efficacy due to the small absorption cross section of chromophores. However, the fluorescence intensity can be improved by a plasmonic particle, by locating molecules at an optimum distance from the metal [124,125]. 

Regarding the diagnostic use of gold-based nanomaterials, many techniques and methods have been developed in the last decades. Among others, the sol particle immunoassay (SPIA) is very interesting [126], and it is based on two kinds of evidence: (i) the sol absorption spectrum does not vary a lot upon biopolymer adsorption on the individual particles; (ii) the sol’s red color changes to red when the particles have a distance under 1–10 times the size of their diameter. An optimized version of this method used AuNPs and monoclonal antibodies on various antigen sites. The AuNPs’ ability to aggregate upon interaction with proteins becomes the basis for the quantitative colorimetric determination of proteins [127]. A new version of the SPIA method using microtitration plates, an ELISA reader, and colloidal gold-trypsin conjugates was proposed for the detection of proteins [128,129].

Spherical AuNPs, AuNRs, AuNSs, and AuNCs, HaAuNPs and AuNcages are the most amazing gold-based nanomaterials for biomedical applications due to: (1) the long body circulation times; (2) the selective accumulation at target sites through an enhanced permeability and retention (EPR) effect or through a specific surface modification; (3) the large absorption in NIR for PTT; and (4) the easy surface functionalization that allows for the drugs delivery. 

The concept of theranostic AuNP nanocomposites emerged for gold-based nanomaterials and combines the functionalities of both contrast and therapeutic agents within a single object. AuNPs, AuNRs, AuNSs and AuNcages were used as theranostic agents because they combine imaging and therapeutic roles [30,76,100,129].

The use of gold-based nanomaterials for a targeted drug delivery, such as anticancer agent, anti-inflammatories, and antibiotics, is highly promising [130,131]. As antitumor agents, delivered by AuNPs, paclitaxel, methotrexate, sulfonamide, 5-fluorouracil, platinum and copper complexes, tamoxifen, herceptin, and doxorubicin are deeply studied [132,133,134,135,136,137,138,139,140,141]. The drug can be conjugated on a gold surface by simple physical adsorption or by using linkers. The effect of conjugates was assessed both in in-vitro models, using tumor cell cultures, and in vivo, generally using mice with induced tumors of different natures and localizations. Moreover, the active molecules anchored on the gold surface can allow for the improvement of the penetration of the complex into the target cells. Multimodal delivery systems were also proposed: the gold-based nanomaterials are loaded with active compounds, such as target molecules, dyes for photodynamic therapy, several therapeutic agents and so on. In general, the drug loading protocol aims at a high loading efficiency through noncovalent electrostatic, π–π stacking, hydrogen bond, and hydrophobic interactions with the carrier surface. In many research investigations, drug molecules have been conjugated with the porous surface of carrier materials or inside a void space, to acquire further increased drug-loading and a specific stimuli-responsive ability [76,77,78]. On these bases, the future drug-delivery systems can be specifically designed and developed, providing us with new perspectives in personalized nanomedicine.

Gold-based nanomaterials and their composites are deeply studied in order to develop specifically optical detectors for biological interactions [120]. In fact, the optical properties of metal nanoparticles are utilized in the design and realization of biochips and biosensors. Numerous kinds of sensors have been developed in the last decade, based on colorimetric and refractometric methods, electrochemical and piezoelectric measures, and certain other methods [117,120,121]. These are of enormous interest in the biomedicine and biosensor field, for examples in the determination of nucleic acids and proteins, in drug screening, in antibodies analyses, in diagnostics, in chemistry and in environmental monitoring. Because the nanoparticle optical response is affected by the particle shape and dimension [127], the interparticle distance [128], and the optical properties of the local environment [129], these became the key parameters: their design and manipulation allow for the sensor’s “tuning” control. These materials are the innovative plasmon resonance biosensor systems (SPR biosensors) in which a bio-specific interaction can be converted into an optical signal.

In the following paragraphs we present the main strategies and methods of the chemical preparation for the various morphologies, with strengths and weaknesses, for the various biomedical applications.

### 2.1. Gold Nanospheres

**Figure 3:**
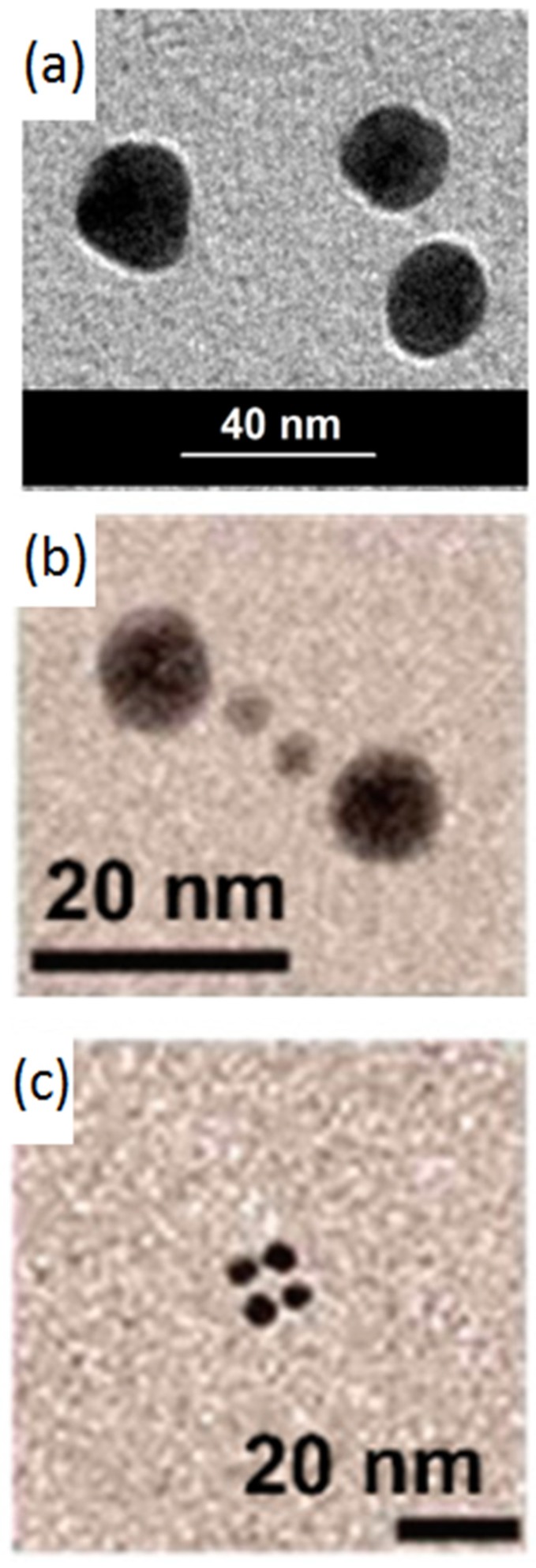
TEM images of AuNPs: (**a**) AuNPs with a diameter of 20–10 nm. Adapted from [153]; (**b**) Self assembled AuNPs with a dimension of 5–15 nm, functionalized by DNA. Adapted with permission from [154]. Copyright (2010) American Chemical Society; (**c**) Self assembled AuNPs with a dimension of 5 nm, functionalized by DNA. Adapted with permission from [154]. Copyright (2010) American Chemical Society.

Many methods for synthesizing AuNPs by chemical, physical and biological processes, using both the bottom-up and top-down approaches have been investigated. A well-known chemical method for synthesizing AuNPs by a bottom-up approach is a wet reduction in the presence of a capping agent, which produces AuNPs with a tunable dimension, easily investigable by Transmission Electron Microscope (TEM) (see Figure 3a). Capped AuNPs are prepared by using a water solution of tetrachloroauric acid as the gold precursor, ligand molecules and a reducing agent such as, for example, sodium boro idrure. Several funtionalizations can be introduced on the gold surface using different strategies: (i) the method of ligand exchange; (ii) bifunctional thiols; (iii) the presence of many different capping agents during synthesis; and (iv) the derivation in a post synthesis process [142,143]. The ligand exchange is a simple and economic way to introduce the ligand on the gold surface, but frequently leads to a partial surface functionalization. In fact, this approach is based on an equilibrium reaction associated with the Nernst distribution. Kluenker et al. [138] show that the surface coverage with the desired ligand depends on the (i) repeated exchange reactions with a large ligand excess, (ii) the diameter of AuNPs, e.g., the surface curvature, and (iii) the ligand steric hindrance. This approach allowed the gold nanoparticles to be used as sensors, for example for food adulterants [144]. The use of bifunctional thiols can in turn be distinguished in two situations according to the functionality that remains toward the outside of the particle. In the case of dithiols, a thiol functionality remains outside, which can in turn bind a particle, due to a high sulfur-gold affinity. In this way, networks of particles connected by organic molecules are easily created [56,145]. In the case of thiols having different functions as a terminal group (acid, amine, ester, ether), this group remains available to interactions with the external environment and to possible functionalizations or links after the synthesis [57,146]. The choice to use many capping agents at the same time is a simple and effective solution to introduce many superficial funtionalities, but requires a severe control of the experimental parameters (concentrations, temperature, and time) to guarantee a material reproducibility. In the case of biomedical applications, where the environment of use is mostly watery, hydrophilic functionalities are mostly preferred, such as acid, amine, ester, and alcohol [57]. 

Moreover, recently, a great challenge has been AuNPs’ spatial organizations and their self assembly. In fact, their distance and orientation produce the electronic and optical coupling between the NPs and modify their plasmonic properties. Chemical approaches, such as a covalent connection using a bifunctional thiolic ligand or electrostatic coupling [58,147,148], have a low repeatability and low yields. Physical approaches have been developed, such as Langmuir-Blodgett techniques [143] and electron beam lithography [144], but they are very expensive and time-consuming. The biological approaches, based on the use of DNA materials, can be easy used in the construction of self assembled nanosystems, and the results are very promising. In fact, the oligonucleotides-templated AuNPs nanostructures can be produced via a complementary base-pair interaction after the surface modification of AuNPs, inducing the assembly of multicomponent frameworks (see Figure 3b,c). In general, the linear AuNPs’ dimeric or trimeric conjugates were attained through AuNPs’ modification by thiol-terminated oligonucleotides, which rapidly became the most general strategy, widely used in research on the DNA-based self-assembly of AuNPs. This technique can, in a precise way, control the interparticle distance, and it is able to produce discrete numbers of DNA-AuNP conjugates [149,150,151,152,153,154]. 

The spherical AuNPs and their composites are widely applied in therapy, diagnostics, drug/gene delivery and sensors [39,70,73,85,100,106,108]. For example, a NIR-triggered, controlled-release system, based on Au/silica core–shell nanospheres, is prepared to study the release of urokinase plasminogen activators. This nanosystem shows two interesting features: a controlled uPA release for reducing side effects, and a locally hyperthermia-enhanced thrombolysis for decreasing the drug dosage [70]. Although AuNPs used in these applications have many advantages, they also have various limitations. Typically, they are limited by accumulating in specific regions, and the efficiency of an antitumoral action on a metastatic cancer cell is relatively low.

### 2.2. Gold Nanorods

The modulable sizes make AuNRs active in the NIR, and this is very interesting in biomedicine. The anisotropic shape, such as a rod, induces two plasmon bands. The first is a transverse plasmon band corresponding to an electron oscillation along the short axis of the rod, at around 520–550 nm. The second is a longitudinal plasmon band, in the range 800–1200 nm.The large use of AuNRs as biomedical therapeutic/imaging agents is due to this property, and the nanorods’ aspect ratio can be tuned by the strict control of the experimental parameters during the chemical synthesis. Furthermore, the presence of chemical or biological analytes can induce the aggregation, disaggregation or change of the local refractive index, which consequently generates changes of the LSPR band, and all this can be used in chemical sensing. 

**Figure 4:**
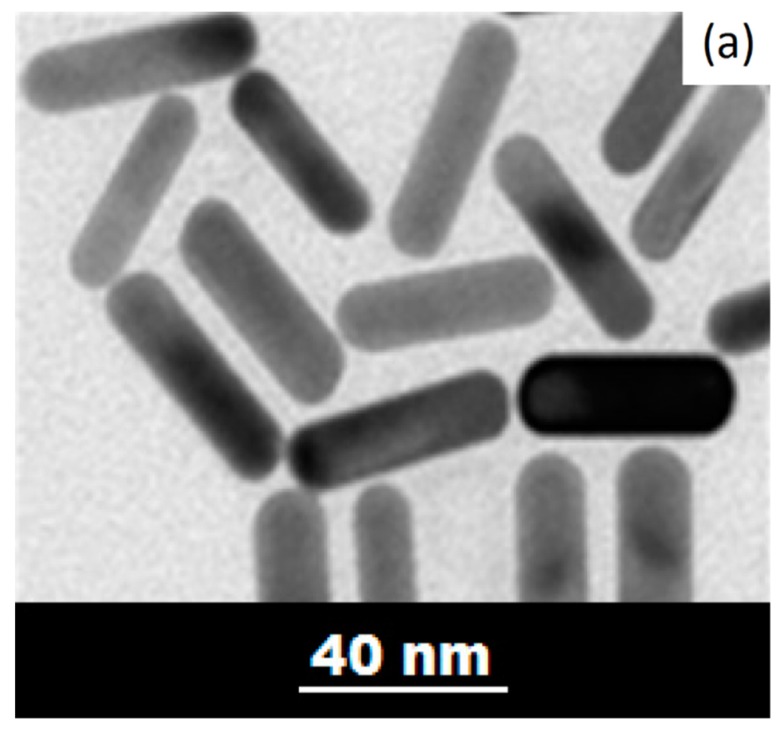

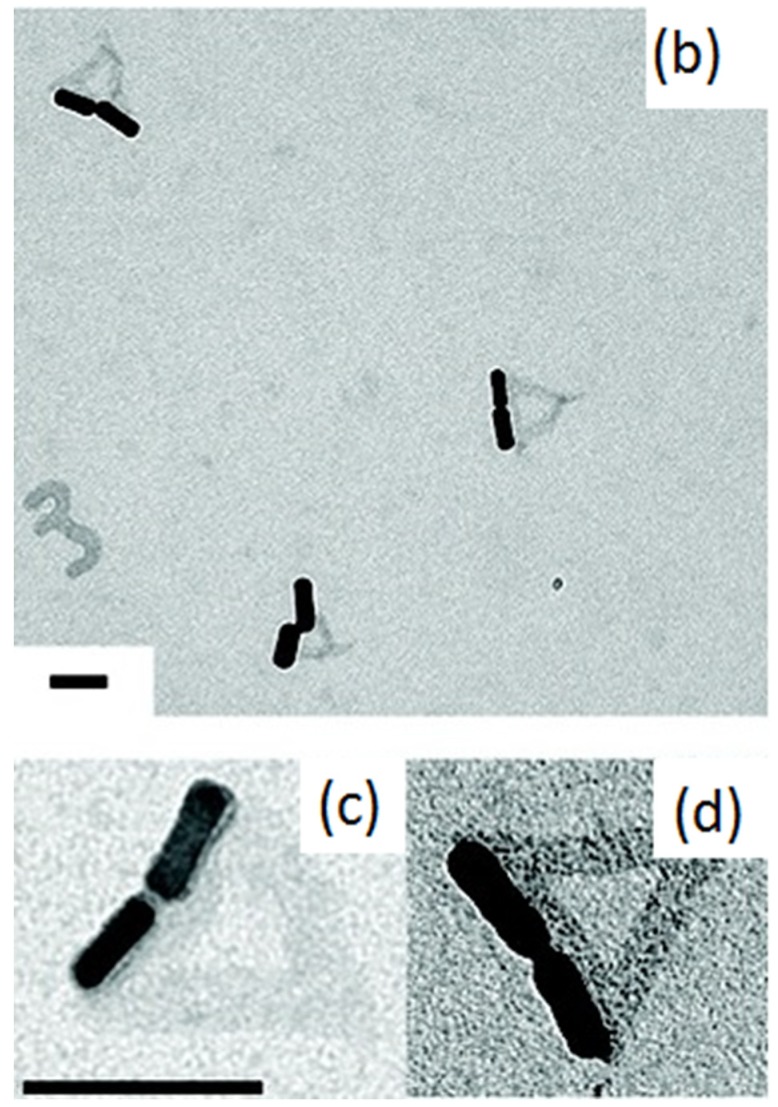
TEM image of self-assembled AuNRs: (**a**) Self assembled AuNRs with a dimension of 10–40 nm. Adapted from [153]; (**b**) Self assembled AuNRs with a dimension of 5–50 nm, functionalized by DNA. Adapted with permission from [157]. Copyright (2011) American Chemical Society; (**c**) Self assembled AuNRs with a dimension of 5–50 nm, functionalized by DNA. Adapted with permission from [157]. Copyright (2011) American Chemical Society. The scale bars in the (**b**,**c**) images are 100 nm.

Regarding the mechanism of AuNRs formation, the discussion is open: a general mechanistic model has not yet been found, although it would be very helpful for defining a synthetic pathway for each nanostructure. AuNRs synthesis includes both thermodynamic and kinetic controls, which increase the parameters number that should be taken into account. In fact, a spherical nanoparticle can be defined only by the average diameter, while the AuNRs’ description involves the aspect ratio, the length, the thickness, the shape yield and the reduction yield. A high control degree of the growth process is necessary, especially when the aim is an accurate control of AuNRs’ aspect ratio. The TEM images of AuNRs are reported in Figure 4. Among several technique to prepare AuNRs, the most diffuse is the seed-mediated colloidal growth methods, where nucleation is achieved separately and the seeds are successively added to the nanorod growth solution. This synthetic procedure does not require specialized equipment, and it has straightforward scalability: important requirements for a clinical translation. It is worth mentioning that there are some methods called “seedless” but that they are commonly characterized by a lower reproducibility and high polydispersity.

AuNRs can be prepared as single-crystalline or as pentatwinned rods, in which the dimensions, surface facets, geometry and composition are different. Pentatwinned nanorods typically have larger dimensions and higher aspect ratios, with longitudinal bands in the NIR range, and they are synthesized by growth on citrate-capped twinned seeds and without silver. Instead, single-crystal AuNRs exhibit smaller dimensions and aspect ratios, the longitudinal plasmon band can be tuned from the visible into the NIR, and they are grown using cetyl trimethylammonium bromide (CTAB) capped single crystal seeds, in the presence of silver nitrate.

In the last decade, the research for the surface functionalization of AuNRs has greatly increased and developed. For example, AuNRs can now be organized in arrays by using oligonucleotides as superficial ligands (see Figure 4). Furthermore, dendrimers and poly(ethylene glycol) (PEG), can be used to substitute CTAB on the AuNRs’ surface, enhancing their biocompatibility, or magnetic nanoparticles can coat their surface, giving magnetic traceability [31,155,156].

The AuNRs’ self-assembly can be induced by DNA, both in one- and two-dimensional structures, using specific experimental conditions: (i) a proper aspect ratio, (ii) DNA concentration, and (iii) electrostatic interaction (between the positively charged nanorod surface and the negatively charged DNA surface). Additionally, ternary compounds can be obtained when the CTAB-coated AuNRs were mixed directly with DNA [10,73]. Another general strategy to achieve biocompatible materials is the functionalization by means of polyethylene glycol (PEG). In fact, PEG-AuNRs exhibited a low cytotoxity, and they were stable in the blood circulation with a half-life time of about 1 h. In general, no accumulation was found in the organs, with the exception of the liver, for at least 72 h. This is a good way to improve nanomaterials’ stability, their bioavaibility and biocompatibility. Recently, reverse micelle-based polyacrylate coating for AuNRs were also proposed with the same aim. The micelle-based coating is a sturdy covering, and it produced hydrophilic AuNRs without modifications in the shape or dimensions. The coating process consists of three main phases. First, in the reverse micelle solution, there are AuNRs and monomer precursors. The in situ polymerization minimized the aggregation and shape change of AuNRs. Second, the polymer concentration was controlled to create a stable covering and to replace the surfactant during the polymerization. As a final point, the reaction was halted before completion so that the cross-linking between the particles could be ignored. This obtained covering could be functionalized and used to adsorb a variety of molecules, such as drugs and dyes, making the AuNRs suitable for biomedical applications. Nanoprobes and nanodevices based on AuNRs will contribute to clinical applications in the near future because these materials show exceptional possible applications.

An important drawback in AuNRs’ preparation is the difficulty in CTAB removing. Various procedures for surfactant elimination were proposed in the literature, and also some CTAB-free procedures. Moreover, pentatwinned AuNRs have a constraint in the shape yield, due to a bad control of the seed production: the development of flow reactors for a reproducible AuNRs synthesis would have an important influence on the industrial approach. Although some microfluidic systems have been projected to improve AuNRs synthesis, there is still a lot of room to reach satisfactory levels of morphological and optical control and modulation.

AuNRs are involved in many biomedical applications, from imaging to therapy to biosensors [2,31,73,120]. For example, CTAB-coated AuNRs were functionalized with polyethylene glycol (PEG) and (BSA), and loaded with an immunoadjuvant imiquimod. This nanoconjugate kills tumors cells under NIR irradiation and activates immune responses in mice metastatic melanoma. Moreover, it induces a long-term antitumor protection [72].

### 2.3. Gold Nanostars

**Figure 5:**
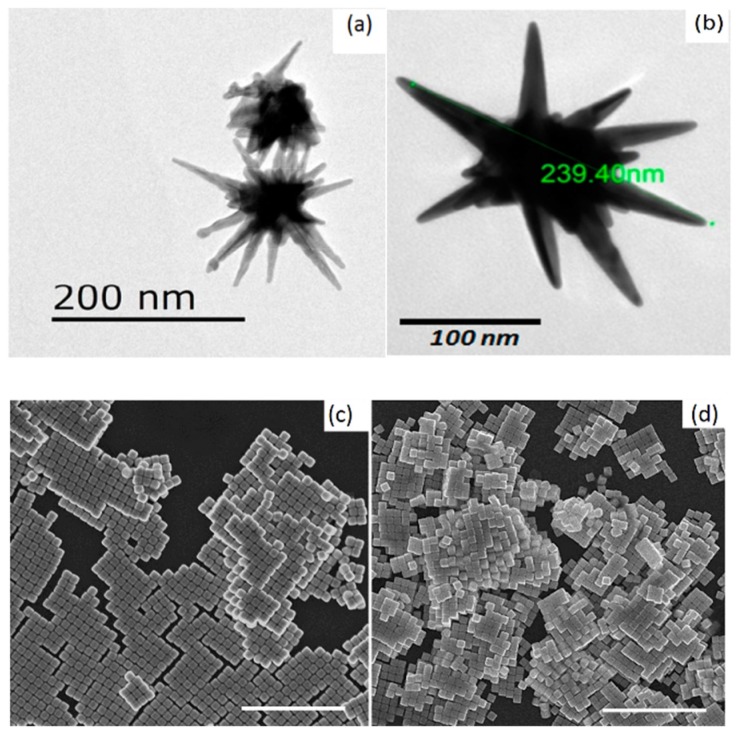
(**a**,**b**) AuNSs TEM images, reproduced and adapted with permission from [161,162] respectively. (**c**,**d**) AuNCs TEM images adapted with permission from [74]. Copyright (2018), American Chemical Society; The scale bars in the (**c**,**d**) images are 1 µm.

AuNSs are gold nanoparticles with sharp branches or tips on their surface (see Figure 5a,b), and can be synthetized in many ways, but in general the protocols are summarized in two main categories: (i) seed-mediated and (ii) one-pot methods [158]. 

The first way is the most used and involves the CTAB, polyvinylpyrrolidone (PVP) N, N-dimethylformamide (DMF) solution and hydrazine. This is the main drawback of these methods, because for these compounds the substitution of the capping agent is generally difficult, and furthermore the post-synthesis functionalization for successive applications is problematic. Some researcher groups have addressed this problem by using zwitterionic lauryl sulfobetaine (LSB) surfactant, which is significantly easier to remove than CTAB [91,159]. The AuNSs can also be fabricated by seeded growth through a two-step surfactant-free approach. In this case, quasispherical seeds were overgrown by adding ascorbic acid as a reductant in the presence of silver ions. By varying the seed dimensions, the final AuNSs’ sizes can be varied from 40 to 200 nm while retaining the star-like morphology with sharp tips, maintaining a SPR tunability from 600 to 950 nm. 

The one pot methods offer the use of the same compound, such as the reducing and stabilizing agent. Recent studies show that the green chemical compounds, such as *N*-2-hydroxyethylpiperazine-*N*-2-ethanesulfonic acid (HEPES), can be used both as a reducing and stabilizing agent. In this one-pot synthesis strategy, the nuclei are evolved in producing nanocrystal seeds and finally nanoparticles. Using piperazine in HEPES, a branches formation on the nanocrystals can be induced. There are also surfactant-free procedures, and, clearly, in these cases the post-synthesis purification is easy, but the nanostars are polydispersed in shapes and dimensions. Another notable problem is the low reproducibility due to the high sensibility to variations of the experimental condition, such as the temperature, concentrations and pH. 

In some methods, AgNO_3_ is added with HEPES to support the growth process of the AuNSs branches: the AuNSs became less polydispersed, but this method is relatively speaking much slower in comparison to the seeded methods that were reported. In fact, it takes over 30 min for the AuNSs synthesis to be completed. The AuNSs that are produced are smaller compared to the seeded ones, thereby having an LSPR peak (which is size dependent) under 630 nm, making them minimally NIR-sensitive. A surfactant-free seedless one-pot method for the AuNSs synthesis, which had the advantages of both the seeded and seedless strategies, was also developed. These seedless AuNSs were easy and rapid to synthesize, with a controllable branch growth. The absorption peak of AuNSs was above 650 nm, with a size of approximately 59 nm. This improves the sensitivity in blue-shifted LSPR. The AuNSs exhibited a good stability when capped with PVP as a stabilizer. When studied for plasmonic colorimetric sensing using glucose as a model analyte, they revealed great stability in ionic environments and sensitivity in detection. This suggests that they are appropriate transducers for biosensing applications. 

Moreover, the AuNSs are prominent in numerous research fields, such a as catalysis, PTT and biosensing. For example, seedless-AuNSs modified with 6.25 × 10^−4^ g mL^−1^ graphene oxide (GOx) were observed to blue-shift relative to control PVP-coated seedless-AuNSs, and the maximum OD of the GOx-modified seedless-AuNSs shifted from 653 to 631 nm (Δ = 22 nm), without a peak broadening, indicating the non-aggregation of the AuNSs. Additionally, AuNSs potentially allowed both hyperthermal treatments and a fluorescent signal by two-photon luminescence (TPL). One should note that the larger AuNSs (>80 nm) are better internalized in cells than the small ones (40–50 nm), despite the latter being optimal for AuNPs: the dimensions surely play an essential role in the nanoparticles/cells’ take-up processes. It is also important to note that the conclusions obtained for a specific morphology could be useless for nanoparticles of the same material but having different shapes [160].

### 2.4. Gold Nanocubes

The anisotropy of AuNCs allows for improved signals based on the local field enhancement at the tips and corners. The TEM images of the AuNCs are reported in Figure 5c,d. Many efforts were done in the new AuNCs designs, in view of the different aims and applications, but a fine regulation is still necessary regarding the quality and reproducibility [163]. The synthesis of AuNCs is commonly considered to be difficult, since the cubes are confined by six closed 100-planes, requiring precise growth settings for their formation. The variations of the experimental parameters, such as the precursor concentrations, mixing conditions, temperature and reaction time, can lead to different results [164].

Microfluidic platforms can be a strategy to solve these issues. Several reproducible protocols based on the microfluid technique were recently proposed [92,165]. They generate homogeneous AuNCs with modulable geometrical dimensions, high yields of the chosen shape, and a low dispersity. The synthesis method is based on three steps and can be transferred into microfluidic techniques, consisting of continuous and segmented flow approaches. Compared to the poor mixing qualities of the conventional batch methods, the microfluidic approaches allow a precise control and mass transfer of the nucleation and grow processes. The success of the protocol depends on a strict schedule of time steps. As result, the microfluidics strategy increases the AuNCs production and reproducibility. Since the shape of the nanocrystal strongly affects the RI (refractive index) the sensitivity, sharp edges, corners and tips further enhance the local electrical field and should have a significant impact on the sensitivity of the nanoparticles. In a comparison of the bulk sensitivities of AuNCs and spherical AuNPs, using equal dimensions (80 nm spheres and 78 nm cubes), the Au spheres have, with 104 nm/RIU (refractive index units), only about half of the sensitivity of the AuNCs, who have 202 nm/RIU, although the lateral dimensions are similar [92]. Therefore, the high impact of edges and corners is demonstrated, and consequently so are the potentialities of AuNCs for sensing applications. 

Furthermore, the AuNCs can be considered appropriate models for the calculation of theoretical sensitivities as one class of anisotropic particles with a simple geometry and homogeneous size distribution. Many computational tools have emerged to model the optical properties of gold nanostructures. Among others, the Discrete Dipole Approximation (DDA) is a powerful technique to calculate the absorption and scattering cross sections of nanostructures of arbitrary shape, structure, and composition. It can be used for a single particle and for assemblies of particles that are surrounded by a medium with a complex dielectric function. Many research groups used DDA to simulate the optical properties of metallic nanostructures and in particular of AuNCs and their ordered 2-D array with different configurations. DDA can be employed to simulate AuNCs’ absorption spectrum.The simulation results show that: (i) the contribution to the extinction cross section comes mainly from the absorption spectrum when the width W ≤ 80 nm; (ii) when one further increases the width, the contribution of the scattering cross section becomes dominant; (iii) the position of its plasmonic band is red-shifted linearly with the side length; (iv) the distribution of the polarization on the corner of the nanocube increases the separation between the negative/positive charges, leading to a red shift in the band position of the LSPR mode, when compared to a gold spherical particle of the same size; and (v) the absorption spectrum of AuNCs exhibits the excitation of single plasmonic band, when compared to the excitation of several plasmonic bands in the case of a silver nanocube. 

To date, the use of structures having sharp nanoscaled corners and edges, such as nanocubes, is a promising strategy to improve SERS performances. In fact, the ‘hot spots’ created at the gaps and junctions between two or multiple adjacent nanoparticles are at the basis of an amplified Raman signal. The nanocubes amplify the antenna-like behaviour to produce an electromagnetic field enhancement with additional 2–3 orders of magnitude. AuNCs can be chemically assembled to create stronger and more reproducible SERS signals: these organized systems can be used in the SERS analysis of protein, as example. A method that does not need the modification of the protein results from the combination of two main ideas: (i) the choice of AuNCs with controlled architectural parameters and (ii) their assembly into organized 2D-arrays featuring a distribution of nanoholes that allow protein entrapment and detection. In the next years one can expect a wide application of these SERS systems in studies of protein–ligand and protein-drug interactions, as well as the identification of physiological mechanisms with several pathological conditions. Moreover, AuNCs show amazing optical properties, such as a high photoluminescence (PL) quantum yield, which is about 200 times higher than that of AuNRs, and which allows them to be used in cell imaging [91].

### 2.5. Gold Hollow Nanoparticles and Nanocapsules

In many applications, such as for example catalysis, cosmetics, photonics, and energy, the AuHNPs show the role of fillers or rheological modifiers. Their effect can be completely evaluated in terms of overall properties, such as the density, volume fraction, dimension and shape. In fact, the void space in hollow structures can be efficaciously used to capture specific molecules, such as chemicals, drugs, cosmetics, enzymes, and then to successively provoke a controlled release. Similarly, the void space has been used to tune the refractive index, increase the active area for catalysis, and advance the skill to tolerate cyclic volume modifications. Due to these features, the AuHNPs are now inducing a great commercial responsiveness. This section is focused on the main works about AuHNPs and AuNcaps, their preparations, characterizations and uses. 

In particular, for AuHNPs, an overview of synthetic approaches is presented, but to keep the review down to a practical level, porous structures and the nanotubes are excluded. The synthetic approaches for preparing AuHNPs can be generally divided into: (i) conventional hard templating syntheses, (ii) sacrificial templating syntheses, (iii) soft templating syntheses, and (iv) template-free methods. 

Conventional Hard Templates. The use of templating against hard particles is conceptually clear and involves a hard template preparation, template coating and selective template removal to obtain hollow structures. The most frequently employed hard templates include polymeric and silica nanoparticles. 

*Sacrificial Templates*. The template is also a reactant in the synthetic process for the shell material formation (or its intermediate). The process is certainly more efficient, because in this case the sacrificial template is totally consumed during the shell-formation. 

*Soft Templates*. The templating against soft templates has attracted great attention, because the hard templates show intrinsic problems, such as low product yields and a low structural hardiness of the shells, as well as a problematic refilling of the hollow interior with guest species. The commonly used soft templates are emulsions, surfactants, micelles, polymeric aggregates/vesicles, and gasbubbles. 

*Template free methods*. One-step self-templated methods based on original mechanisms, such as inside-out Ostwald ripening, have been proposed to synthesize AuHNPs. A possible mechanism in two iterative steps, inside-out Ostwald ripening was also suggested: (i) during the initial step, amorphous solid nanospheres are created and their surface layer crystallizes, firstly due to contact with the surrounding solution; (ii) the materials inside the solid spheres dissolve successively; all of this gives the driving force for the spontaneous inside-out Ostwald ripening. The interior process of particle spontaneous dissolution can result from a surface layer stabilization by the surfactants and, as a result, the interior materials will have a relatively high surface energy and will therefore dissolve preferentially.

A relatively new family of hollow inorganic nanoparticles include the gold nanorings and AuNcages. 

The colloidal gold nanorings can be easy fabricated by the galvanic replacement of sacrificial cobalt nanoparticles (CoNPs) in an Au^3+^ ion solution, in the presence of PVP as a stabilizing agent. The PVP molecular weight, Au/Co ratio and CoNPs dimension are the experimental parameters that control the modulation of the outer/inner diameter. These materials showed distinctive SPR properties, due to structural differences, with a red shift of λ_max_ at 653 nm, in view of the nanoring’s external dimensions being about 40 nm. 

**Figure 6:**
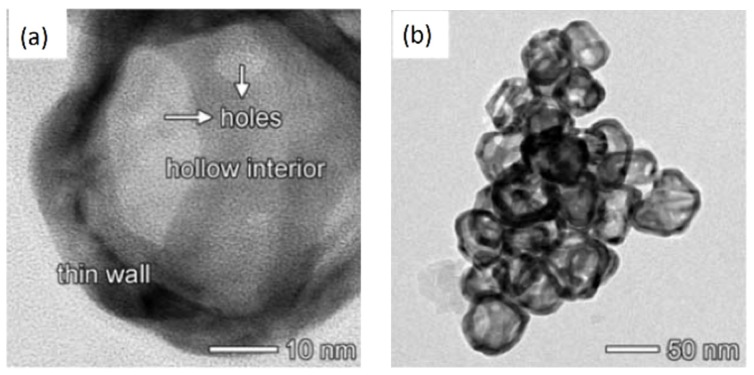
(**a**,**b**) TEM images of gold nanocages; reproduced and adapted from [166].

In a similar way, the galvanic replacement of silver nanocube templates can produce AuNcages. In particular, three Ag atoms are replaced by one Au atom, bringing about the gradual formation of various porous alloy structures, i.e., AuNcages, shown in Figure 6. In this case, the LSPR band shifts from 430 to 440 nm for the cubes to 700–900 nm for the AuNcages.

In general, when a liquid/solid core, for example a cavity filled with a drug, is surrounded by a film, the structure is considered a capsule. In fact, in AuNcaps a thin film gives a protective coating and delays the release of active components. They mainly consist of a controlled release, drug bioavailability improvement and drug toxicity mitigation. AuNcaps can be easily administered intravenously, reaching the target and releasing the encapsulated drug.

Hollow inorganic nanoparticles could be used as imaging and diagnosis agents. In fact, AuNcages are also used as an optical imaging contrast agent for optical coherence tomography (OCT), and they can be tuned to strongly absorb the NIR, allowing for PTT applications [76,78]. As an example, a novel multifunctional nanoplatform based on hyaluronic acid-modified Au nanocages (AuNCs-HA) was developed. This nanoplatform had three functionalities: (i) an excellent LSPR peak in the NIR region, useful for photoacoustic (PA) imaging and PTT; (ii) a high-energy rays (X-ray) absorption and auger electrons generation, useful as a radiosensitizer for radiotherapy; and (iii) a good photocatalytic property and large surface area, useful for photodynamic therapy (PDT). This AuNCs-HA platform allowed the combination of radiotherapy and phototherapy, inducing a complete tumor growth inhibition [78]. Other applications involve sensing; for example, AuNcages were used to modify a carbon ionic liquid electrode for the sensitive detection of luteolin in a sensing platform [166].

## 3. Key Properties for Biomedicine

Nanotechnology research is endorsing the investigation and improvement of stimuli-responsive materials, both for the creation of model systems to recognize their response, and in designing and implementing *smart* nanomaterials with a stimuli-responsive structure, composition and functionality.

Engineered gold-based nanosystems are promising in this field because they can be carried out with a wide range of chemical functionalities that are post-synthetically easily modified. These features allow them to react to specific external stimuli, such as the pH, temperature, redox potential and light, with optical and electronical modifications, solvent absorption and aggregation capacity. 

**Figure 7:**
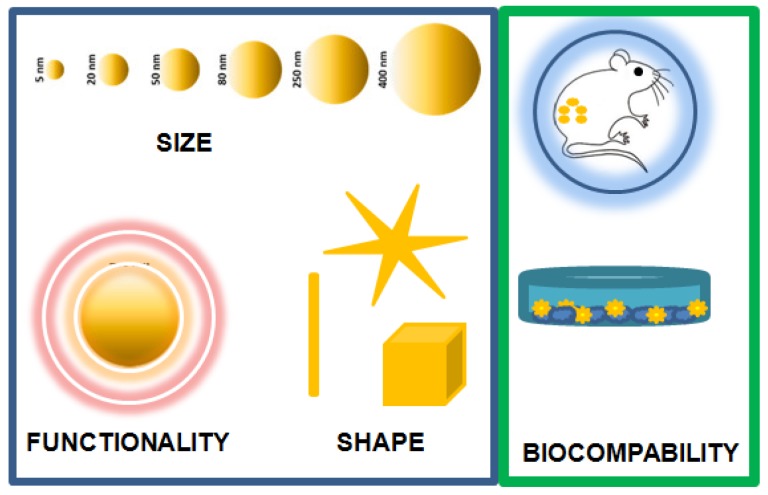
Key properties of engineered gold-based nanomaterials in biomedical applications.

The strict control of their key properties, schematized in Figure 7, such as the size, shape and surface features, directly affects the smart performance and requires specific synthetic methods.

As we have already seen in the above paragraphs, by means of a chemical synthesis it is now possible to vary the sizes, functionalizations and forms of gold nanomaterials, and one can even manage to induce biocompatibility. Furthermore, the characterization of these materials has evolved, allowing an increasingly detailed and in-depth study that is aimed at defining the structure-property relationship more clearly. The main characterization techniques, fundamental for engineered-gold nanomaterials investigations, are UV−vis−NIR, SERS spectroscopies and TEM. It is important to realize that these are complementary to each other, and that all could be used together in all cases. 

The shape, size, and structures of gold-based nanomaterials strongly determine the rates of absorption and scattering, as well as the position of LSPR. In general, AuNPs have an LSPR band in the range 500–600 nm, more or less broad depending on the polydispersity. AuNRs have two plasmonic bands, a strong longitudinal band in the near-infrared region and a weak transverse band in the visible region. One can note that the position and intensity of the AuNRs’ LSPR peaks are unusually more sensitive to the local refractive index and local dielectric environment than similarly sized spherical nanoparticles, which gives the basis of their use as colorimetric probes or biosensors [2,31,73,120]. AuNSs typically show an LSPR band of the core and multiple plasmon bands corresponding to the tips and core-tip interactions [156,157,158,159]. The AuHNPs and AuNCs have the highest sensitivity factors, and their LSPR bands are modulable throughout the visible and into the NIR regions by varying their dimensions. 

The AuNPs’ ensemble produces the SERS effect, originated by the strong amplification of electromagnetic fields. An extensive enhancement occurs at the AuNPs’ surface, because the intensity of the Raman signal depends on the fourth power of the local electric field, which is very high at the AuNPs surface, due to the plasmon resonance. This enhancement also originates from electromagnetic coupling, due to a charge transfer between the AuNPs’ surface and the adsorbed molecules. The two main strategies for the SERS detection are the direct identification of Raman-active adsorbed molecules and the indirect detection of molecules that are incorporated into a biolabel. When a molecule is localized on a non-spherical gold nano-object, its Raman scattering signal is enhanced by the contributions of the absorptions in the NIR and the extremely high electric field intensities at their tips or in the hollow structure. This can also be exploited for accurate information on the surface functionalization of a general gold nano-object.

The optical spectrum can tell an expert eye a large amount of information, such as the shape, dimension and dispersity. On the other hand, to obtain the particle dimensions with a high accuracy, a TEM image can be provided. However, if a fair reproduction of the three-dimensional morphology is mandatory, electron tomography should be employed. It is also important to understand that a single TEM image is not the best way to verify the essence and quality of the nanocompounds because during drying a shape segregation can occur, and the product will largely accumulate on a particular area of the grid. One should consider that in order to acquire sufficient TEM statistics for the shape and size, several images must be analysed, preferably at different magnifications. Finally, the preparation method of TEM specimens also merits attention; longer drying times frequently produce a better organization of the nanomaterials on the grid. A small-angle X-ray scattering (SAXS) also allows us to estimate the dimension of nanomaterials, such as nanorods, directly from the solution, analysing billions of particles, and if complemented with small-angle neutron scattering (SANS) it is also possible to investigate the ligand layer surrounding the particles. 

Moreover, nuclear magnetic resonance (NMR), Surface Enhanced Raman Scattering (SERS) and the X-ray photoelectron spectroscopy (XPS) are extremely useful techniques to study the functionalized surface [55,57,58]. NMR spectroscopy allows one to characterize gold nanomaterials (including the dimension evaluation, surface chemistry functionalization and ligand density) thoroughly by one- and multiple-dimensional NMR and diffusion-order NMR spectroscopies. Raman spectroscopy is a non-destructive detection technique, providing information on the molecule structure and vibration. However, in general, the signal intensity of Raman scattering is low with respect to the background noise. The presence of gold nanoparticles significantly enhances the signal intensity of Raman scattering up to 100–110 times. This effect is due to the electromagnetic enhancement based on surface plasmon excitation, and to the charge transfer between the adsorbed molecule and the metallic surface. Therefore, AuNP-based SERS nanosensors are used in biochemistry sensing and tumour detection [167,168]. The XPS method gives exhaustive information on the chemical composition of a solid surface and on the electron state of the surface elements. The shape, dimension and layered structure of nanoparticles influence the XPS data in several ways, such as the peak intensities and relative peak intensities, peak binding energies, and background signals from electrons that have lost energy. Depending on the sample and the analysis aim, one can extract information about nanomaterials from each of these data.

Indeed, gold nanomaterials confirm a strategic application in cancer diagnostics and therapeutics. Despite this, it is necessary to evaluate the unintended side effects on human health. Many individual studies investigated the cytotoxicity, toxicity, bioaccumulation, retention time, and physiological response of several different gold nanomaterials [169]. On the other hand, the literature shows that many contradictions and incoherent information has resulted on the concrete effects of nanoparticles. Moreover, the consequence and reaction of the biological system to the administration of gold nanoparticles are difficult to systematically probe, mainly due to the heterogeneity of human cells and tissues. The general knowledge is that the properties of gold nanoparticles, such as shape, size, surface chemistry and targeting ligand, strongly influence their toxicity [50,51,52,53,54]. Furthermore, the oxidation state of gold is very important. AgNPs are very toxic, because of their relative ease of oxidation to Ag^+^ salts, but despite the fact that Au^0^ is much more difficult to oxidize than Ag^0^, its oxidizability always depends on the NP size, shape, and surrounding ligands. In all biological organisms the redox reactions are intrinsic, and the potential oxidation of Au^0^ atoms to toxic Au(I) or Au(III) ions (which could subsequently be leached) should not be underestimated. More than the isotropic ones, the anisotropic AuNPs present these potential risks because of their highly exposed AuNP surface areas and defects. However, the toxicity often depends on the dose, and there is no standard dose known to be either toxic or safe. Finally, the purity of the gold nanomaterial formulation has a key a role in the toxicological investigations: free metal ions and/or surfactants, present in the solution, could be the cause of toxicity. As a consequence of this, clinical applications of gold nanomaterials are currently controversial. 

The development of generally applicable methods to evaluate the biocompatibility of gold nanoparticles and to have a fine understanding of their interaction with a living system is still necessary. 

## 4. Prospective Outlooks and Conclusions

Engineered gold-based nanomaterials used in biomedicine hold great potential in therapy, diagnostics, drug/gene delivery, and sensors. Their key properties, such as size, shape and surface features, directly affect their smart behavior and their applications. In the next years, for Alzheimer’s disease, HIV, hepatitis, tuberculosis, diabetes mellitus, and other diseases, new diagnostic applications for gold nanomaterials will be predictable. It is well known that nanogold conjugates are exceptional tools for bioimaging, and that they can be improved by using various optical technologies, including confocal laser microscopy, resonance scattering dark-field microscopy, and two-photon luminescence. Moreover, they have been found to be applicable to bioanalytical studies such as immunoassays. Nevertheless, challenges remain open with regards to several aspects, such as: (i) the precise characterization of molecular targets; (ii) biocompatibility; and (iii) ensuring that these molecules only affect targeted organs. In fact, the main difficulties that contribute to a low efficiency in all fields of applications (therapy, drug/gene delivery, diagnostic and sensing) can be summarized in the low concentrations that reach the active site and the very short residence time in the cellular and anatomical sites. These challenges require common efforts by researchers to improve and develop novel engineered nanomaterials. Moreover, nowadays investigations are focused on increasing the effectiveness and, at the same time, on decreasing the toxicity. We can therefore conclude that engineered gold nanomaterials, while still showing limitations within clinical uses, remain the most promising and amazing materials in biomedicine applications.

## Figures and Tables

**Table 1:** Morphologies, dimensions, and functionalities of selected gold nanomaterials used in several biomedical applications.

Morphology	Size (nm)	Functionality	Biomedical Investigations	Ref.
**Nanospheres**				
	20	acid	cytotoxicity test	[48]
	90	acid & amine	optical sensors	[4,70]
	10	sulfonate	drug delivery	[49,59]
	10	sulfonate	fluorescence resonance energy transfer (FRET)	[62]
	100	glycol	photothermal therapy (PTT)	[32]
	5	acid & amine	computed tomography	[35]
	20	ester	sensor	[86]
	10	sulfonate	sensor	[36]
	10	alginate	sensor	[23]
**Nanorods**				
	16–50	acid	cytotoxicity test	[48]
	16–50	ether	cytotoxicity test	[48]
	100	acid & amine	optical sensor	[8]
	16–50	acid & amine	optical sensor	[2]
	38–118	glycol	dye fluorescence enhancement	[10]
	15–60	amine	conjugation with *Cytochrome C*	[28]
	25–35	acid & amine	dye absorption enhancement	[18]
**Nanostars**				
	18–70	acid & ester	cell transfection	[81]
	50	amine	singlet oxygen production	[82]
	40	HEPES-aptamer	anticancer effects	[87]
		peptides-PEG	photothermal properties	[88]
	30	lauryl sulfobetaine	photothermal properties	[89]
**Nanocubes**				
	10–300	gold/prussian blue analogue	biosensor	[4]
	80	acid	biosensor	[90]
	5–10	acid	catalyst	[24]
	100	amine	imaging	[91]
	50–80	amine	biosensor	[92]
**Hollow nanoparticles**				
	80–100	pyridinium	drug delivery	[77]
	200–1000	acid & ester	drug delivery	[79]
	50	acid & ester	photothermal properties	[84]
**Nanocapsules**				
	40	acid & ester	optical sensitivity	[75]
	50	acid	photothermal properties	[76]
	30	dye	imaging	[78]
